# Photonic Crystal Optical Tweezers with High Efficiency for Live Biological Samples and Viability Characterization

**DOI:** 10.1038/srep19924

**Published:** 2016-01-27

**Authors:** Peifeng Jing, Jingda Wu, Gary W. Liu, Ethan G. Keeler, Suzie H. Pun, Lih Y. Lin

**Affiliations:** 1 Department of Electrical Engineering, University of Washington, Seattle WA, 98195, USA; 2 Department of Bioengineering, University of Washington, Seattle WA, 98195, USA

## Abstract

We propose and demonstrate a new optical trapping method for single cells that utilizes modulated light fields to trap a wide array of cell types, including mammalian, yeast, and *Escherichia coli* cells, on the surface of a two-dimensional photonic crystal. This method is capable of reducing the required light intensity, and thus minimizing the photothermal damage to living cells, thereby extending cell viability in optical trapping and cell manipulation applications. To this end, a thorough characterization of cell viability in optical trapping environments was performed. This study also demonstrates the technique using spatial light modulation in patterned manipulation of live cell arrays over a broad area.

Methods for interacting with biological samples, including single cells, have made great progress in the past couple of decades, revealing high-resolution details on microscopic size-scales. Of particular importance, vibrating micro-electro-mechanical systems (MEMS) resonators possess impressive sensitivity to the mechanical properties of single cells and bio-molecules, resolving properties^1^,^2^,^3^,^4^,^5^,^6^,^7^ including mass, density, size and stiffness. Detection is accomplished through observation of resonant-frequency shifts induced by mass additions or changes in attached bio-samples. While promising high measurement sensitivity, these devices are hindered by unpredictability in sample placement, since the resonant frequency is affected by both the added mass and its position, thereby causing a loss of measurement precision. To remediate this problem, optical trapping has a unique opportunity for non-invasive sample manipulation and control that could aid these resonant devices^8^ in obtaining precise measurements. By optical position-fixing, long-term monitoring of single cells and their physical properties is possible. Such measurements can enable critical biological studies, including consideration of cell-growth size dependencies or unregulated growth applicable to understanding cancer mechanisms or those of other diseases^9^. This relation between mass and cell growth is a fundamental question for biologists, and high-resolution, high-precision measurement can have great potential in medicine and drug discovery.

Optical trapping has become a widely utilized, non-invasive tool for manipulation in biological applications, to place, identify and modify live cells^10^,^11^, nano-particles and DNA strands^12^. However, photodamage to cells limits measurement duration and its application in the life sciences. To address this shortcoming, methods have been developed for increased trapping efficiency – the capability to trap particles measured through trap stiffness and minimum trapping intensity, and thus trapping at a lower-intensity: optoelectronic tweezers (OET)^13^, plasmonic optical tweezers^14^,^15^ and photonic crystal (PhC) waveguides^16^. Nonetheless, these techniques are not readily compatible with MEMS. OET accomplishes large-scale parallel manipulation with two electrodes to achieve low-intensity optical trapping and avoids the photodamage effect, but integrating OET fabrication methods with current MEMS resonators is not straightforward. Plasmonic optical tweezers use highly localized light intensity to increase the trapping force; although used to trap living cells, such as yeast cells^15^ and *Escherichia coli* (*E. coli*)^17^, the large amount of heat generated by the plasmonic surface prohibits long-term manipulation of living cells. PhC waveguides also highly localize light energy and trap particles through evanescent waves; however, the waveguide is not easily coupled with vibrating MEMS structures, and its nano-size trapping cavity is not versatile for various eukaryotic cells, whose sizes can vary from 3–10 μm.

To overcome these compatibility challenges, we propose and demonstrate an approach of low-intensity optical manipulation utilizing the interaction of laser light with a two-dimensional photonic crystal (2D PhC). Photonic crystals have become an important structure for manipulating photons and have demonstrated great success and potential for various applications. Beyond the efforts made on PhC geometry design, they have been utilized in light-trapping and manipulation devices as optical waveguide resonators^16^, optical fibers^18^ and PhC slabs^19^,^20^. Efficient and enhanced optical trapping for small particles has been demonstrated in both the near field^16^,^21^ and far field^22^,^23^. Our method differentiates itself from other PhC trapping approaches by illuminating the laser light on top of the PhC structure, thus requires no critical light coupling. Furthermore, it enables optical trapping with reduced photodamage by minimizing the required laser intensity, while still maintaining the same trapping capability when manipulating living cells over broad areas (~500 μm^2^) under the microscope using 20x objective lenses. This technique can be easily integrated with lab-on-chip systems and potentially applied to measuring the mass of single living cells^8^. In such an application, the proposed method has the added benefit of trapping particles at the PhC substrate surface with the trap pattern determined by the PhC design. By integrating the PhC structure with a MEMS resonant device for mass detection, better vibrational coupling is achieved between the trapped cell and the MEMS resonator which is necessary for mass sensing since the trapped particle needs to move with the vibrating MEMS resonator.

In considering the applicability of optical trapping in long-term cell manipulation and its suitability for the proposed applications, it is essential to understand how optical energy affects the cell over time. Although researchers have used the previously noted methods to manipulate living cells, insufficient viability measurements of living cells have been reported, while existing cell lifetime research for optical traps used only *E. coli* bacteria cells^24^,^25^,^26^,^27^. These investigations focused more on optimizing wavelength to reduce optical damage, achieving ~10 minute lifetimes at a ~1100 nm wavelength using an objective lens with a high numerical aperture (N.A. = 1.2) and high laser intensity in the specimen plane^24^. Therefore, experimental research in this area on eukaryotic cells with optical tweezers is not fully explored. Moreover, even though laser wavelength can be optimized, the photodamage is still a severe limit for long-term biological study of living cells.

In our experiments, a simple optical setup was used to guide a loosely focused laser beam onto the surface of a 2D PhC, which then modulates the profile of the laser beam and generates a confined trapping area above the surface of the 2D PhC. We achieved prolonged cell viability (~30 min), confirming that the 2D PhC uses less power while sustaining the same cell trapping force. We also demonstrate higher trap stiffness by trapping polystyrene beads and *E. coli* cells, and these experimental findings were consistent well with finite-difference time-domain (FDTD) simulation results. The highly localized intensity in our method has been a general concern for viability of cells, and this concern extends to plasmonic and PhC waveguide trapping methods. However, we experimentally verify that the viability is largely determined by overall intensity, rather than localized intensity.

## Simulation and Experimental Results

### FDTD Simulation

**Figure 1:**
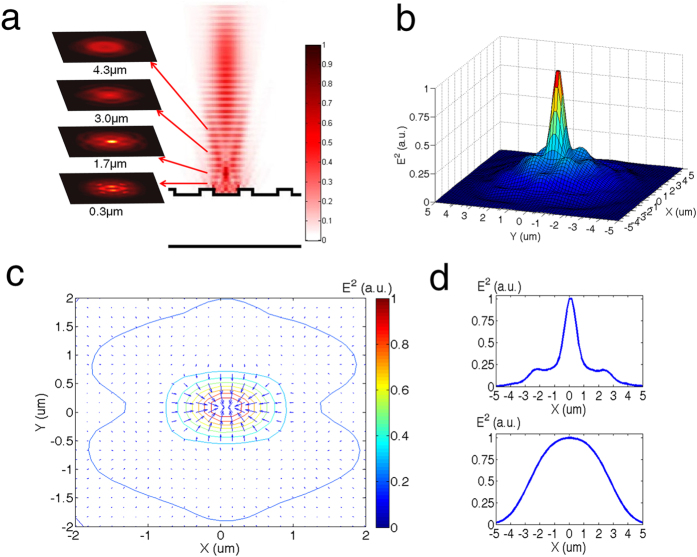
FDTD simulation of the laser light field modulated by a 2D PhC, demonstrating enhanced optical trapping through increasing gradient of the intensity distribution. The PhC is a square lattice with a period of 5.8 μm and 3.6 μm-diameter holes. (**a**) Vertical cross-section of the optical energy density and corresponding horizontal cross-sections at 0.3, 1.7, 3.0 and 4.3 μm above the surface of the 2D PhC. (**b**) 3D surface plot of the optical intensity at the trap location, ~1.67 μm above the surface of the PhC surface. (**c**) Contour and gradient of the optical intensity at the trap plane. (**d**) Comparison of the trap potential between the PhC-modulated light field and the Gaussian profile in traditional laser traps.

Our proposed method entails designing the PhC structure to modulate the incident optical field and to create efficient optical traps. The PhC comprises a square lattice with a period of 5.8 μm and 3.6 μm-diameter holes. Various hole depths were simulated, and 500 nm was found to be optimum to avoid confining the optical energy inside the holes. This optimized depth also works well with most of the cells in our experiments. After being reflected by the 2D PhC, the phase of light is modulated. The configuration of the optical traps is generated by light modulation and therefore depends on the dimensions of the 2D PhC. If the depth of the holes in the PhC is much greater than the hole diameter, most of the energy is confined inside of the features of the PhC and the particles are trapped by evanescent waves^19^,^20^,^21^. However, if the holes are shallow, most of the modulated light is scattered back into free space and generates an efficient trap positioned above the surface of the 2D PhC. We performed simulations of the modulated light field produced by a PhC with shallow holes using the FDTD method through the open-source software package MEEP^28^. Figure 1a shows the optical energy density distribution of the modulated light field, presented by both vertical and horizontal cross-sections at different heights. The vertical cross-section shows that the modulated light field generates a focused volume for the optical trap located at about 1.67 μm above the surface of the 2D PhC. The four images on the left side of Fig. 1a are horizontal cross-sections at a height of 0.3, 1.7, 3.0 and 4.3 μm above the PhC surface. Figure 1b shows a three-dimensional shaded surface plot for the horizontal cross-section of the intensity distribution at the trap location. The contour and gradient of the intensity at this height is shown in Fig. 1c. The asymmetry of the gradient along the x- and y-axis is caused by the light polarization. Because the trapping force is proportional to the gradient of the light intensity distribution 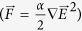, Fig. 1c also indicates the enhanced trapping potential generated by the PhC-modulated light field. This is further elaborated by Fig. 1d, which shows a comparison between trap potentials (proportional to the intensity distribution) in traditional laser trapping without a PhC and that above the surface of the 2D PhC. The top diagram is the normalized energy density along the *x*-direction of the PhC-modulated light field, and the bottom diagram is the distribution for traditional laser trapping. By increasing the gradient of the intensity distribution, enhanced trapping force is generated above the 2D PhC surface.

Because the incident laser beam is affected by the trapped particle, a comparison of trap enhancement is performed with and without a 2 μm-diameter polystyrene sphere above the PhC. The result in supplementary Fig. 2 shows that the trapped particle above the surface further enhances the optical trap through a slight focusing effect. To investigate the possibility of trap enhancement due to the effect of the standing wave caused by interference of the incident and reflected light, we also used a Gaussian pulsed-laser beam as the light source to eliminate interference. The FDTD simulation result (supplementary Fig. 3) shows a similar light confinement effect, suggests that the enhancement is mainly caused by the reflected light field modulated by the PhC. The shape of the modulated light field is mainly determined by a single hole, while interference from the periodic PhC structure further enhances the intensity of the modulated light. The FDTD simulation result comparing the modulated optical intensity profile from a single hole to that from a PhC structure is shown in supplementary Fig. 4.

### Two-Dimensional Photonic Crystal Optical Trapping

**Figure 2:**
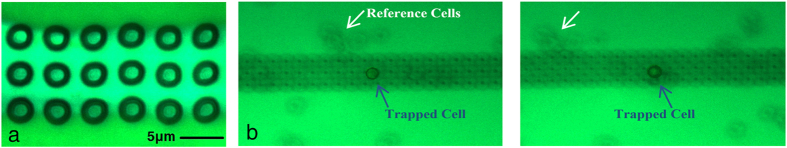
(**a**) An optical microscope image of the 2D PhC. (**b**) Images of yeast cells being trapped (left) and dragged (right) on the surface of the 2D PhC.

The utility of the proposed method was first verified using eukaryotic yeast cells, which are typically 3-4 μm in diameter. We used a Nd:YVO_4_ 1064 nm continuous wave (CW) laser to trap cells with a 50X objective lens (N.A. = 0.55). The details of the optical setup are described in the Online Methods section. Figure 2a shows an optical microscope image of the 2D PhC structure, and the snapshots in Fig. 2b show dynamic trapping and dragging of a yeast cell along the 2D PhC surface. Fabrication of the PhC is described in the Methods section. A 1.2 μm layer of parylene-C was deposited on top of the PhC platform to create a thin, transparent polymer film that protects and better planarizes the PhC surface.

**Figure 3:**
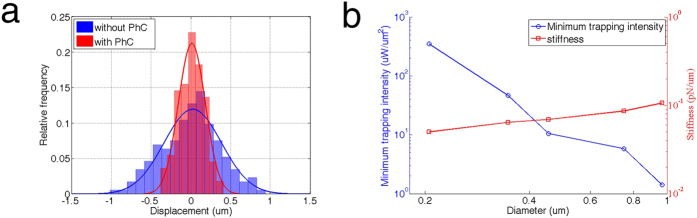
Optical trapping efficiency characterizations. (**a**) Relative frequency histograms of ramdom movement for trapping 750 nm polystyrene beads at a 5.8 μW/μm^2^ laser intensity with (red) and without (blue) the 2D PhC platform. The stiffness are 0.156 pN/μm and 0.030 pN/μm, respectively. (**b**) Minimum trapping intensity (blue) and stiffness (red) for different sizes of polystyrene beads at the laser intensity of 0.35 mW/μm^2^.

To characterize the trapping efficiency, the stiffness and minimum trapping intensity were measured by trapping polystyrene beads of sizes ranging from 0.2–1 μm. Stiffness is defined as 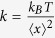, where 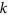 is the stiffness, 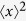 is the position variance of the trapped particles, *k*_*B*_ is the Boltzmann constant and *T* is temperature. The minimum trapping intensity is measured under microscope. The intensity is the average intensity determined by dividing the optical power under the microscope objective lens by the area of the Gaussian beam. A single particle is trapped at higher power, and the power is reduced until the particle escapes from the trap. The lowest power and intensity are then recorded as the minimum trapping power and intensity. Figure 3a shows the relative frequency histogram of the Brownian motion of a trapped 750 nm polystyrene bead under 5.8 μW/μm^2^ laser intensity with (red) and without (blue) the 2D PhC platform. The beads are more firmly trapped above the 2D PhC platform, and their motion distribution is narrower than the distribution without the 2D PhC, Theses findings are consistent with the FDTD simulation results. The histograms follow Gaussian distributions, allowing calculation of the stiffness by the equipartition method^16^. The trap stiffness is obtained by characterizing the displacement histogram under a specific incident optical intensity. The stiffnesses were 0.156 pN/μm with PhC and 0.030 pN/μm without PhC. The stiffness is theoretically calculated to be 0.25 pN/μm with PhC based on FDTD simulation results. This is higher than the experimental result due to the parylene layer above photonic crystal which has a higher refractive index than water and therefore reduces the intensity of the modulated light field after reflection. We expect the measured trap stiffness to become higher if no covering layer or materials with lower refractive indices are used. Figure 3b shows the minimum trapping intensity required to confine the beads and the stiffness measurement for various sized polystyrene beads. Two sets of data are shown: the measured minimum intensity for trapping polystyrene beads with sizes ranging from 0.2–1 μm; the trap stiffness measured at an intensity of 0.35 mW/μm^2^ (minimum intensity to trap the 0.2 μm beads) above the surface of the 2D PhC for the polystyrene beads (red curve). The polystyrene beads were initially trapped with higher intensity above the center of a hole in the 2D PhC. The intensity was then decreased, and the minimum trapping intensity value was recorded when the trap released the beads. The minimum trapping intensity in Fig. 3b is significantly lower than with conventional optical tweezers, which typically requires ~1 mW/μm^2^ 13.

We then performed similar measurements using *E. coli* bacteria, which are typically 0.5 × 2 μm rod-like cells. Using traditional optical tweezers, the measured stiffness was 0.0095  N/μm, while on the 2D PhC platform the measured stiffness was 0.088 pN/μm, resulting in an enhancement factor ~10.

### Cell Viability Characterization

**Figure 4:**
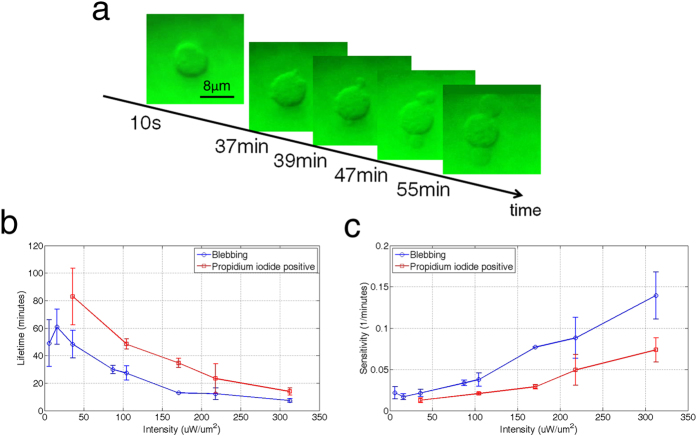
Cell viability characterizations. (**a**) Status of NIH/3T3 mammalian cells under 36.0 μW/μm^2^ illumination. The cell began blebbing after 30 minutes. (**b**) Lifetime measurements of theNIH/3T3 cells. The blue and red curves represent the times until blebbing and PI staining occurred, respectively. (**c**) Sensitivity of mammalian cells, Defined as 1/min of the lifetimes reported in (**b**).

Although reports have proposed a linear relationship between *E. coli* viability and laser power^25^, a systematic characterization of eukaryotic cell viability under optical trapping has not yet been performed. Given the promise of our developed platform for optical trapping and manipulation of live cells, we aimed to measure the viability of live NIH/3T3 mammalian fibroblast, yeast, and *E. coli* cells under optical trapping. To measure viability, we suspended cells in media containing propidium iodide (PI) (PBS+1% BSA+2 μg/mL PI). PI is a commonly used cytofluorometric indicator to identify apoptotic and necrotic cells. As PI is cell membrane-impermeable, this dye is excluded from live cells. However, during cellular apoptosis or necrosis the cell membrane loses its selectivity, and PI is able to permeate the cell and intercalate between DNA bases. PI binding induces an emission spectrum shift from 535 nm to 617 nm and enhances PI fluorescence 20- to 30-fold^29^. Therefore, apoptotic and necrotic cells may be identified by PI (red) staining. We employed a single-blind method to evaluate cell viability. One scientist set the laser power while another scientist, blinded to the power value, observed and recorded the cell lifetime. The 1064 nm near-infrared CW laser was used to trap cells under a 50× objective lens, and a mercury lamp was used to excite the PI dye during the experiments. Figure 4a shows the time-dependent morphology of a trapped NIH/3T3 cell under 36.0 μW/μm^2^ laser intensity. The cell maintained its morphology for more than 30 min before blebbing, as seen by the protrusion from the main cell body. Blebbing of mammalian cells in damaged cells correlates with cells death from either apoptosis or necrosis^30^, and was used as a preliminary indication of cell death. Figure 4b shows the measurement results of NIH/3T3 cell lifetime versus PI staining. The time until blebbing (blue curve) and when the cell stained positive for PI (red curve) was observed at each of the laser intensities. As shown in Figure 4b, blebbing occurred before the cell stained positive for PI, indicating that the PI dye may be constrained by diffusion into the cell and bind with the DNA; therefore, in these experiments PI staining was not an immediate indicator of cell necrosis. As evident from the trends in Figure 4b, the time interval between blebbing and PI staining was not constant and was dependent on the optical power.

Sensitivity is used as another parameter to quantify photodamage to optically trapped cells and is defined as the reciprocal of the cell lifetime^24^. Figure 4c shows the sensitivity of mammalian cells based on the measurement results in Fig. 4b. The sensitivities using both cell blebbing and PI staining lifetimes exhibit an approximately linear relationship with the laser intensity, which is consistent with the results reported by Neuman *et al.* using *E. coli* cells. We also performed the same characterization on *E. coli* cells, and the results are shown in supplementary Fig. 5. The sensitivity flattens at low laser intensity and approaches 20 minutes in lifetime, which matches the replication time of *E. coli* cells.

**Figure 5:**
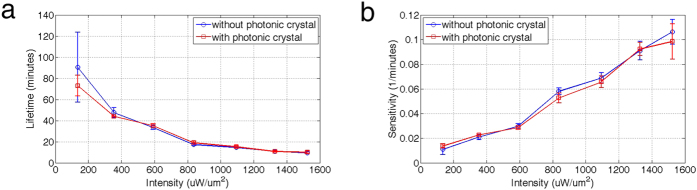
Effect of highly localized optical fields on cell viability. (**a**) Lifetime versus optical intensity for trapping yeast cells with and without the 2D PhC platform. (**b**) The corresponding cell sensitivity to light intensity. The results show that the cell viability is primarily determined by the overall optical intensity, not the localized intensity.

One question in enhanced optical trapping through highly-localized optical fields is whether the effect of photodamage depends on the localized intensity even if the overall intensity is lower. To answer this question, we performed viability measurements of the trapped cell versus incident optical intensity with and without the PhC platform. Figure 5 compares the lifetime and sensitivity of yeast cells under both trapping methods. The blue curves represent trapping with traditional optical tweezers (without PhC), and the red curves represent trapping on the 2D PhC platform. The two curves are very similar, suggesting that the localized high-intensity light above the surface of the 2D PhC does not compromise cell viability.

**Figure 6:**
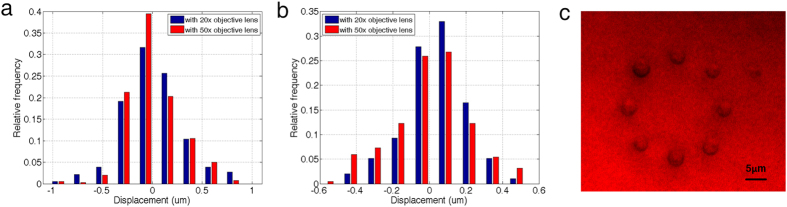
(**a**) Histogram of displacement for trapped 964 nm polystyrene beads under 20X and 50X objective lenses with a 28 μW/μm^2^ laser intensity, with stiffnesses of 0.04 pN/μm and 0.06 pN/μm, respectively. (**b**) Histogram of displacement for trapped *E. coli* under 20X and 50X objective lenses with a 28 μW/μm^2^ laser intensity, with stiffnesses of 0.09 pN/μm and 0.15 pN/μm, respectively. (**c**) An example of optical trapping with yeast cells using a pre-programed SLM.

One advantage of the proposed method over plasmonic optical tweezers is that the locations of the optical traps are not at predetermined “hot spot”. As the incident beam moves, the trapped particle moves with the beam as shown in Fig. 2. To further demonstrate this versatility and the potential of parallel manipulation over a broad area on the 2D PhC platform, we changed the objective lens to 20X (N.A. = 0.22), performed optical trapping and measured the trap stiffness on the 2D PhC. Figure 6a shows the relative frequency histogram of Brownian motion for trapped 964 nm-diameter polystyrene beads, and Fig. 6b shows the relative frequency histogram of motion for trapped *E. coli* bacteria. The same measurements were performed using the 50X objective lens, and the results were compared. The trap stiffness appears to be unaffected by the objective lens magnification, indicating that particles can be trapped with loosely focused light under a larger field of view of the microscope. As an example, Fig. 6c demonstrates parallel manipulation of yeast cells using a spatial light modulator (SLM).

## Conclusion

In this work, we demonstrate a novel optical trapping method for live eukaryotic and bacteria cells utilizing a 2D PhC. As shown by both FDTD simulation and experimental results, modulation of the incident light field by the PhC structure produces a trapping volume above the 2D PhC that enhances the trapping force and prolongs the trapping duration of live cells. By thoroughly quantifying the viability and sensitivity of live cells when trapped by traditional optical tweezers and the proposed methods, we experimentally verify that cell viability is mainly determined by the overall optical intensity instead of the highly-localized optical intensity produced by this new method.

We also demonstrate that this straightforward optical system could easily enable large-scale trapping under microscope by using smaller numerical aperture lenses, while still maintaining a similar trapping efficiency with the reduced light intensity. Being compatible with lab-on-chip systems, such as MEMS resonators, this method could have significant impact on cell control and manipulation capabilities thereby enabling numerous applications and improving measurement accuracy for biological studies.

## Methods

### Two-dimensional photonic crystal fabrication

The photonic crystal (PhC) was fabricated in silicon due to its high-dielectric properties and its well-established processing techniques. Using photolithography, a PhC feature pattern was transferred to a photoresist etch mask. The pattern had a square lattice orientation consisting of circular holes with diameters of 3.6 μm and a periodicity of 5.8 μm. The holes were etched using a parallel-plate reactive-ion etcher (RIE) to about 500 nm in depth.

The process stripped the photoresist and chemical-vapor deposited a 1.2 μm layer of parylene-C to create a thin, transparent polymer film that protects and better planarizes the PhC surface in addition to giving adequate refractive index contrast with silicon. Parylene-C is also bio-compatible and provides conformal encapsulation of the device.

### NIH/3T3 mammalian cell culture

NIH/3T3 fibroblasts (ATCC) were cultured according to supplier instructions. Cells were cultured in DMEM+10% FBS and passaged every three days or at confluency with 3 × 10^5^ cells in a T25 flask.

### Yeast cell culture

EBY100 cells were grown in SDCAA media at 30 °C for about 20 h to reach a stationary state before passage into a PBS (pH = 7.4). The genotype of the EBY100 cell strain is described by Chao *et al.*^31^. The sample was centrifuged before experiments, and the fsupematant removed to leave the sediments pellet.

### *Escherichia coli* culture

Strain ER2738 *Escherichia coli* (*E. coli*) were cultured according to supplier (New England BioLabs) instructions. Briefly, a single colony was inoculated into the LB media and allowed to grow overnight. Finally, the bacteria were diluted for the trapping study.

### Propidium iodide staining

For assessing NIH/3T3 cell viability under laser optical trapping, cells were trypsinized, washed and resuspended in a propidium iodide (PI) solution (PBS+1% BSA+2 μg/mL PI) at a density of 1.0 × 10^6^ cells/mL. For assessing yeast and E. *coli* viability under optical trapping, cells were grown overnight with vigorous shaking and diluted 1:1 with a concentrated PI solution for the final dilution.

### Optical trapping on the PhC platform

#### Optical setup

We used a simple optical setup shown in Supplementary Fig. 1 to guide a loosely focused Nd:YVO_4_ 1064 nm laser onto the surface of a 2D PhC through a Zeiss Axio Imager fluorescence microscope. The interaction between the incident light and the PhC generates a diffraction pattern above the surface of the 2D PhC to trap the cells. The center of the laser beam is aligned with the center of a hole in the PhC. We then added a 10 μL specimen droplet onto the 2D PhC and covered it with a glass slide with about 0.03 mm spacing. The optical trapping results were recorded by a CCD camera connected to the microscope.

#### Stiffness measurement

We used Tracker (version 4.84), a freeware video analysis and modeling software, to determine the positions of the trapped particles. The stiffness was calculated by the equipartition method using 
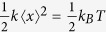
, where 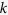 is the stiffness, 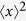 is the position variance of the trapped particles, *k*_*B*_ is the Boltzmann constant and 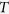 is temperature.

#### Minimum trapping intensity measurement

The polystyrene beads were initially trapped with higher intensity above the center of a hole in the 2D PhC. The intensity was then decreased, and the intensity value was recorded when the trap released the beads.

## Additional Information

**How to cite this article**: Jing, P. *et al.* Photonic Crystal Optical Tweezers with High Efficiency for Live Biological Samples and Viability Characterization. *Sci. Rep.* **6**, 19924; doi: 10.1038/srep19924 (2016).

## Supplementary Material

Supplementary Information
