# Supplementary material for: Photonic Crystal Optical Tweezers with High Efficiency for Live Biological Samples and Viability Characterization
Source: Sci Rep. 2016 Jan 27;6:19924. doi: 10.1038/srep19924 (PMC4728687; doi:10.1038/srep19924)
Supplement: Supplementary Information [file srep19924-s1.pdf]

Supplementary Information

Photonic Crystal Optical Tweezers with High Efficiency for Live Biological Samples and Viability Characterization

Peifeng Jing[1], Jingda Wu[1], Gary W. Liu[2], Ethan G. Keeler[1], Suzie H. Pun[2], and Lih Y. Lin[1]

[1]Department of Electrical Engineering, University of Washington, Seattle WA, 98195, USA
[2]Department of Bioengineering, University of Washington, Seattle WA, 98195, USA

Supplementary Figure

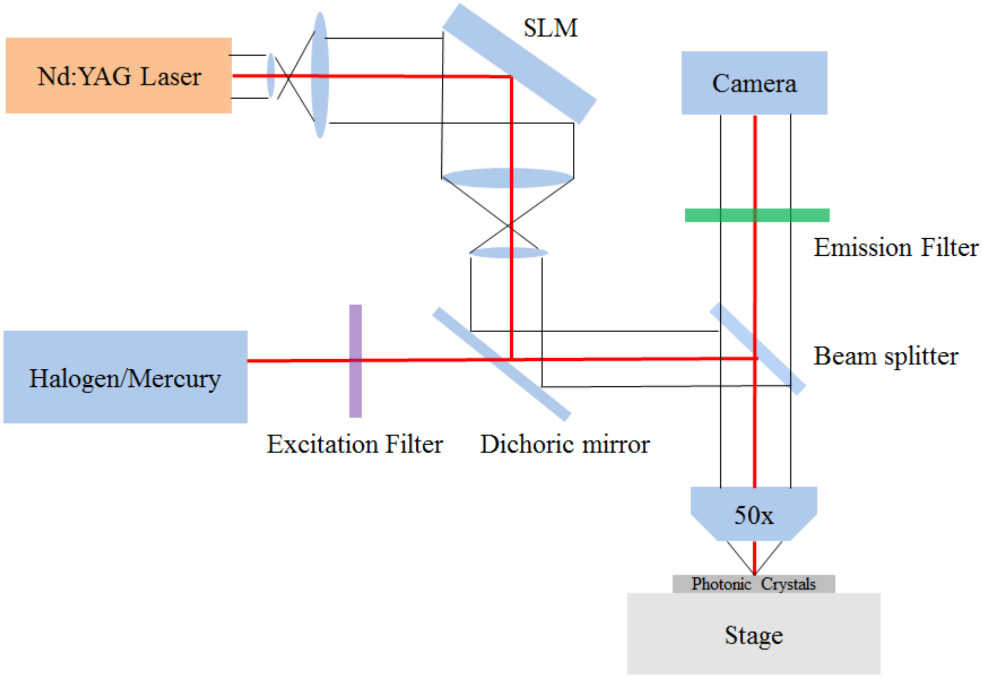

Supplementary Figure 1 | Schematic diagram of the optical system for the photonic crystal optical trapping experiments.

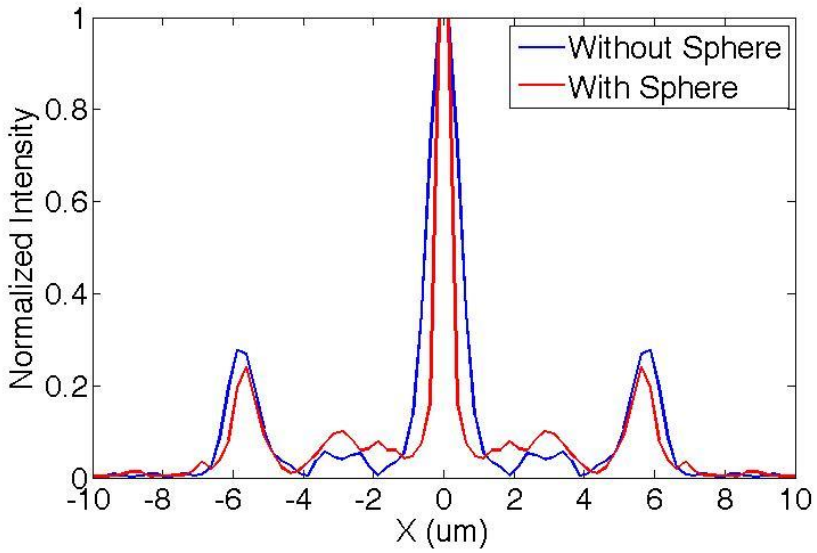

Supplementary Figure 2 | FDTD simulation of comparison between reflected intensity profile with and without a 2μm-diameter polystyrene sphere above the photonic crystal.

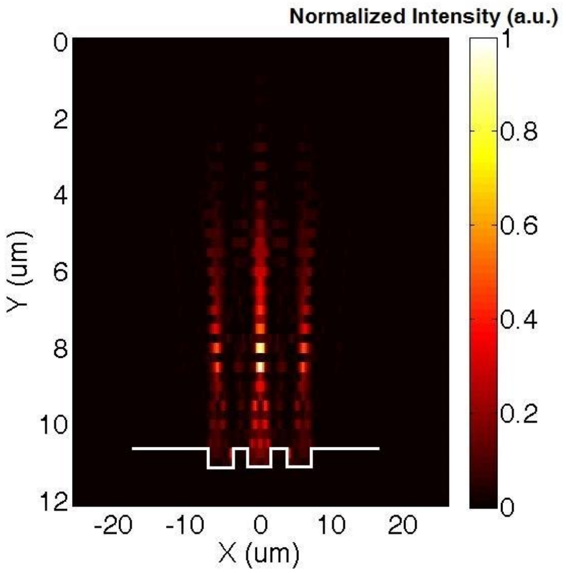

Supplementary Figure 3 | FDTD simulation of 1μm-width Gaussian pulsed laser reflection above a two-dimensional photonic crystal.

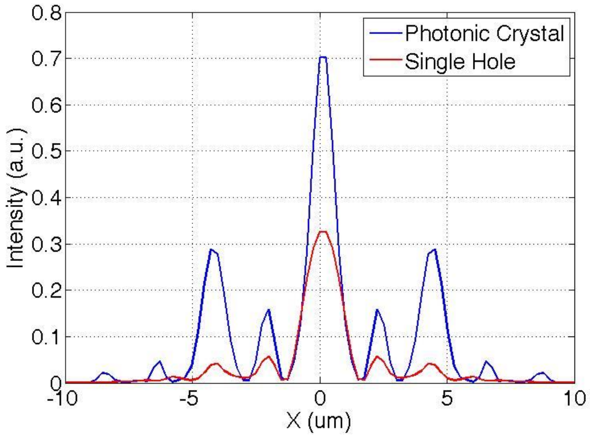

Supplementary Figure 4 | Intensity comparison of the modulated light field. Red line: The laser beam incidents on a single hole. Blue line: the laser beam covers 5x5 holes in a photonic crystal structure.

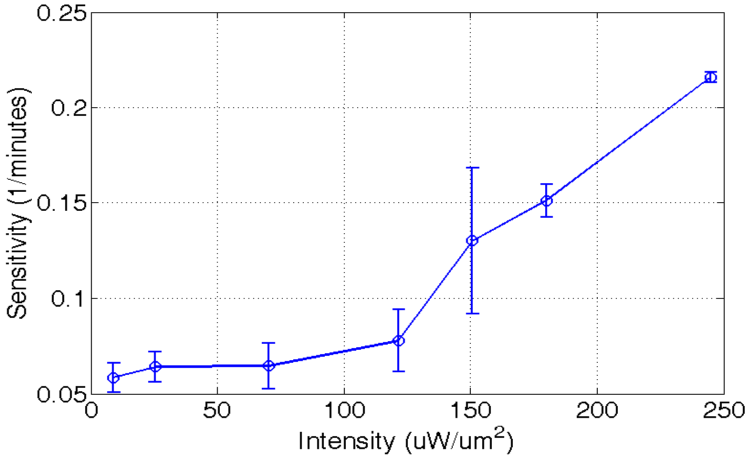

Supplementary Figure 5 | Sensitivity of *E. coli* cells in the PhC optical tweezers. The sensitivity is defined as the reciprocal of the lifetime.
